# Targeted Therapy for Cancers: From Ongoing Clinical Trials to FDA-Approved Drugs

**DOI:** 10.3390/ijms241713618

**Published:** 2023-09-03

**Authors:** Ha Yeong Choi, Ji-Eun Chang

**Affiliations:** College of Pharmacy, Dongduk Women’s University, Seoul 02748, Republic of Korea

**Keywords:** cancer targeted therapy, lung cancer, colorectal cancer, prostate cancer, clinical trials, FDA-approved drugs

## Abstract

The development of targeted therapies has revolutionized cancer treatment, offering improved efficacy with reduced side effects compared with traditional chemotherapy. This review highlights the current landscape of targeted therapy in lung cancer, colorectal cancer, and prostate cancer, focusing on key molecular targets. Moreover, it aligns with US Food and Drug Administration (FDA)-approved drugs and drug candidates. In lung cancer, mutations in the epidermal growth factor receptor (EGFR) and anaplastic lymphoma kinase (ALK) gene rearrangements have emerged as significant targets. FDA-approved drugs like osimertinib and crizotinib specifically inhibit these aberrant pathways, providing remarkable benefits in patients with EGFR-mutated or ALK-positive lung cancer. Colorectal cancer treatment has been shaped by targeting the vascular endothelial growth factor (VEGF) and EGFR. Bevacizumab and cetuximab are prominent FDA-approved agents that hinder VEGF and EGFR signaling, significantly enhancing outcomes in metastatic colorectal cancer patients. In prostate cancer, androgen receptor (AR) targeting is pivotal. Drugs like enzalutamide, apalutamide, and darolutamide effectively inhibit AR signaling, demonstrating efficacy in castration-resistant prostate cancer. This review further highlights promising targets like mesenchymal-epithelial transition (MET), ROS1, BRAF, and poly(ADP-ribose) polymeras (PARP) in specific cancer subsets, along with ongoing clinical trials that continue to shape the future of targeted therapy.

## 1. Introduction

Cancer remains a global health challenge, accounting for a significant proportion of the global mortality rate. Although conventional treatments such as surgery, chemotherapy, and radiation therapy have been the mainstays of cancer management, their limitations in terms of efficacy and tolerability have spurred a search for more precise and effective therapeutic approaches [1]. Targeted therapy has emerged as a promising paradigm in cancer treatment, driven by advances in our understanding of the molecular basis of cancer.

The advent of targeted therapy has brought about a shift towards personalized medicine, wherein treatment strategies are tailored to the unique genetic and molecular characteristics of individual tumors. This approach aims to exploit specific molecular vulnerabilities within cancer cells while sparing normal tissues, thus minimizing side effects and improving patient outcomes. Although targeted therapies hold promise for personalized cancer treatments, therapeutic resistance remains a challenge due to tumor cell plasticity, leading to resistance mechanisms such as target mutations, pathway reactivation, and microenvironment interactions. Tumor heterogeneity further complicates treatment responses. Identifying resistance mechanisms has led to improved clinical outcomes through alternative agents [2].

In this review, we focus on three prevalent cancer types, as follows: lung cancer, colorectal cancer, and prostate cancer. These malignancies are major contributors to cancer-related mortality rates [3], necessitating the urgent need for more effective and tailored treatment options.

In lung cancer, the discovery of driver mutations in genes, such as the epidermal growth factor receptor (EGFR) and anaplastic lymphoma kinase (ALK), has been transformative. These molecular alterations serve as actionable targets for small molecule inhibitors and monoclonal antibodies, leading to marked clinical responses in subsets of lung cancer patients. We examined US Food and Drug Administration (FDA)-approved targeted drugs that specifically inhibit EGFR and ALK pathways, offering insights into their mechanisms of action and clinical implications.

Colorectal cancer, another major malignancy, has witnessed significant advances in terms of targeted therapies. By targeting the vascular endothelial growth factor (VEGF) and EGFR pathways, anti-angiogenic agents and EGFR inhibitors have revolutionized the treatment landscape for metastatic colorectal cancer. We present an overview of these targeted agents, elucidating their role in the management of this disease.

In prostate cancer, androgen deprivation therapy has long been a cornerstone of treatment. With better insights into androgen receptor (AR) signaling and the emergence of castration-resistant prostate cancer, novel agents targeting the AR pathway have emerged. We explored FDA-approved drugs that have shown efficacy in castration-resistant prostate cancer, highlighting their mechanism of action and clinical relevance.

Additionally, we discuss emerging targets in specific cancer subsets, such as mesenchymal-epithelial transition (MET), ROS1, BRAF, and poly(ADP-ribose) polymerase (PARP), that hold promise for further advancing targeted therapies. By summarizing ongoing clinical trials, we shed light on the future directions of targeted therapy for these malignancies.

This review aims to provide a comprehensive overview of the current landscape of targeted therapies in lung cancer, colorectal cancer, and prostate cancer. By compiling key molecular targets and corresponding FDA-approved drugs, we hope to contribute to a deeper understanding of the potential and challenges in the field of targeted cancer treatments. Ultimately, our pursuit of targeted therapies holds promise in terms of improved outcomes and enhanced quality of life for cancer patients.

## 2. Lung Cancer

According to cancer statistics from the American Cancer Society, lung cancer is projected to have the highest mortality rate among both men and women in 2023. Approximately 350 individuals succumb to lung cancer daily, which is nearly 2.5 times higher than the number of deaths attributed to the second leading cause of cancer mortality. Approximately 81% of lung cancer deaths are directly linked to cigarette smoking, with an additional 3% attributed to second-hand smoke [4]. The three-year relative survival rate for lung cancer has improved over time due to advancements in treatments and earlier detection [5,6]. However, the five-year relative survival rate for lung cancer remains relatively low, standing at 23% between 2012 and 2018, which underscores the necessity for novel early detection tools and therapies.

Lung cancer exists in two primary forms, as follows: non-small cell lung cancer (NSCLC) (85%) and small cell lung cancer (SCLC) (15%). Within NSCLC, it is classified into three main types, as follows: adenocarcinoma, squamous cell carcinoma, and large cell carcinoma. Among them, adenocarcinoma is the most prevalent subtype, constituting around 40% of lung cancers [7].

The treatment approach for NSCLC varies by stage. For stage I or II, surgical resection is recommended, if feasible. Non-surgical cases might benefit from conventional or stereotactic radiotherapy. Cryoablation, radiofrequency ablation, and microwave treatment offer potential treatment options post-surgery, post-radiotherapy, or post-salvage therapy, as well as for palliative care in advanced NSCLC [8]. Notably, up to 69% of advanced non-small cell lung cancer (NSCLC) patients harbor molecular aberrations [9], indicating that this is a promising target for molecular targeted therapy. As the understanding of lung cancer biology and genetics continues to advance, the identification of actionable targets has become instrumental in enhancing treatment outcomes and potentially improving survival rates.

These statistics highlight the urgent need for ongoing research and the development of novel therapeutic approaches, particularly in the context of molecularly targeted therapies. Advancements in personalized medicine, and the discovery of innovative targeted agents, hold the potential to transform the landscape of lung cancer treatment, offering hope for better patient outcomes, and ultimately, reducing the burden of this devastating disease.

Targeted therapies for lung cancer have been summarized in Table 1.

### 2.1. EGFR

In NSCLC patients, EGFR mutations are more prevalent among never-smokers, those with adenocarcinomas, females, and individuals with an East Asian ethnicity [10]. The most common sensitizing mutations include exon 19 deletions (del19) and exon 21 L858R point mutations (L858R), which account for 85–90% of EGFR mutations overall, and mutations termed “common EGFR mutations” [11].

First-generation EGFR-tyrosine kinase inhibitors (TKIs), such as gefitinib and erlotinib, have demonstrated enhanced progression-free survival (PFS) compared with chemotherapy; it particularly benefits patients with del19 mutations, females, and never-smokers [12]. However, despite the improved PFS, no significant overall survival advantage has been reported for gefitinib or erlotinib when compared with chemotherapy [13]. Unfortunately, many patients who are initially responsive to first-generation EGFR-TKIs eventually develop acquired drug resistance. Over 50% of lung adenocarcinoma patients who exhibit resistance to gefitinib or erlotinib have been found to develop the T790M mutation, a substitution of methionine for threonine at position 790 in the EGFR kinase domain [14]. Additionally, MET amplification and human epidermal growth factor receptor 2 (HER2) amplification have been observed in patients with acquired resistance to these EGFR-TKIs [15].

Second-generation EGFR-TKIs, including afatinib and dacomitinib, have shown promise in clinical studies. In the LUX-Lung 7 study, afatinib exhibited significantly longer PFS (median 11.0 months with afatinib vs. 10.9 months with gefitinib) and time-to-treatment failure (median 13.7 months with afatinib vs. 11.5 months with gefitinib) compared with gefitinib as a first-line treatment for EGFR mutation-positive NSCLC patients [16]. 

The third-generation EGFR-TKI, osimertinib, is an irreversible inhibitor that selectively targets EGFR-TKI-sensitizing mutations as well as the T790M resistance mutation, thus overcoming acquired resistance to first- and second-generation EGFR TKIs. Osimertinib has demonstrated significantly prolonged PFS and OS compared with chemotherapy in patients who progressed after first-generation EGFR TKI treatment with the T790M mutation [17]. Additionally, osimertinib has been evaluated as a first-line treatment compared with first-generation EGFR-TKIs in EGFR mutation-positive advanced NSCLC patients, where it exhibited a longer PFS (18.9 months with osimertinib vs. 10.2 months with gefitinib or erlotinib), median duration of response (17.2 months with osimertinib vs. 8.5 months with gefitinib or erlotinib), and higher survival rates at 18 months (83% with osimertinib and 71% with gefitinib or erlotinib). The safety profile was similar in both groups, and the osimertinib group showed less frequent serious adverse events [17]. 

However, even with first-line osimertinib treatment, patients have developed both primary (e.g., EGFR exon 20 insertions and BIM deletion polymorphism) and acquired resistance (e.g., EGFR tertiary mutations, loss of T790M, EGFR amplification, MET amplification, HER2 amplification, aberrant fibroblast growth factor receptor (FGFR) signaling pathway, aberrant insulin-like growth factor 1 receptor (IGF1R) activation, anexelekto (AXL) activation, RAS/RAF/MEK/ERK pathway activation, PI3K/AKT/mTOR pathway activation, histological/phenotypic transformation, altered cell cycle genes, and carcinogenic gene fusion) [18].

### 2.2. ALK

ALK is a tyrosine kinase receptor encoded by the insulin receptor family member located on chromosome 2 [19]. ALK rearrangements arise from inversions or translocations on chromosome 2, resulting in the fusion of variable regions from partner genes with exon 20 of ALK. Echinoderm microtubule-associated protein-like 4 (EML4) is the most common fusion partner in the NSCLC ALK rearrangement [20]. EML4-ALK plays a critical role in lung tumorigenesis, which is effectively inhibited by ALK selective inhibitors [21].

ALK rearrangements account for approximately 7% of molecular aberrations in lung adenocarcinomas [22]. These fusions are more frequently found in never/light smokers, adenocarcinomas, those with non-Asian ethnicities, and males [21].

Crizotinib, a first-generation ALK-TKI, targets not only ALK but also ROS1 and MET. In phase I and phase II trials, crizotinib demonstrated a median PFS of 9.7 months and 8.1 months, respectively, in patients with advanced ALK-positive NSCLC [23,24]. In the phase III PROFILE 1007 trial, crizotinib was compared with pemetrexed or docetaxel chemotherapy in advanced ALK-positive NSCLC patients. This study demonstrated the superiority of crizotinib over chemotherapy with a significantly improved median PFS (7.7 months vs. 3.0 months) and objective response rate (ORR) (65% vs. 20%). In addition, crizotinib showed an acceptable safety profile [25]. Another phase III study, PROFILE 1014, was performed to compare the efficacy of crizotinib with standard chemotherapy (pemetrexed plus either cisplatin or carboplatin) as a first-line treatment for advanced ALK-positive NSCLC patients. Crizotinib significantly prolonged the median PFS (10.9 months with crizotinib vs. 7.0 months with standard chemotherapy) and increased the ORR (74% with crizotinib vs. 45% with standard chemotherapy) [26]. 

Despite the superior efficacy of crizotinib, around 20% of patients treated with crizotinib develop ALK-acquired resistance mutations, with L1196M being the most well-known mechanism. Additionally, various ALK-independent resistance mechanisms (e.g., EGFR alteration, mast/stem cell growth factor receptor (KIT) alteration, insulin-like growth factor 1 receptor alteration) have been identified [27]. Most patients treated with crizotinib experience a relapse within 1–2 years, and around 20–30% of ALK-positive NSCLC patients have brain metastases at diagnosis, with the incidence increasing up to 50% during cancer progression [28]. Crizotinib has poor CNS penetration, leading to persistent CNS metastasis during treatment [29].

Second-generation ALK-TKIs, including alectinib, ceritinib, and brigatinib, demonstrate better CNS penetration. An ALEX study compared alectinib with crizotinib as a first-line treatment for untreated, advanced ALK-positive NSCLC patients, showing superior CNS activity and delayed CNS progression with alectinib [30]. Alectinib was also shown to be a potential first-line treatment option when compared with crizotinib [31]. An ASCEND-4 study was performed to evaluate the efficacy of ceritinib as a first-line therapy in advanced ALK-rearranged NSCLC compared with chemotherapy. The median PFS was 16.6 months with ceritinib and 8.1 months with chemotherapy [32]. In the ASCEND-5 trial, the efficacy of ceritinib was compared with chemotherapy in ALK-rearranged NSCLC patients who had previously progressed following chemotherapy and crizotinib treatments. Ceritinib showed a significant prolonged median PFS (5.4 months for ceritinib vs. 4.6 months for chemotherapy), proving that it is the more potent ALK inhibitor compared with chemotherapy after crizotinib failure [33]. Another second-generation ALK-TKI brigatinib also proved to be a promising TKI for patients with ALK-positive NSCLC when compared with crizotinib. The 12 month PFS was significantly higher with brigatinib than with crizotinib (67% vs. 43%) [34].

Various resistance mechanisms for second-generation ALK-TKIs have been identified, including secondary ALK mutations and signaling pathway alterations. G1202R substitution is the most common resistance mutation following second-generation ALK-TKI treatment [35]. Lorlatinib, a brain-penetrant and third-generation ALK-TKI, has emerged as a promising option after progression on second-generation ALK-TKIs. Lorlatinib effectively targets ALK-dependent resistance mechanisms such as L1196M and G1202R substitutions. Due to its ability to penetrate the CNS and cover ALK mutations, lorlatinib has been suggested as a subsequent and potential first-line therapy for ALK-positive NSCLC patients [36]. When compared with crizotinib as a first-line therapy for advanced ALK-positive NSCLC patients, the lorlatinib group showed prolonged PFS and increased intracranial response [37].

### 2.3. MET

The MET receptor tyrosine kinase (RTK), and its ligand hepatocyte growth factor (HGF), have emerged as attractive targets for lung cancer treatment. In lung cancers, MET can be activated through various mechanisms, such as binding to HGF, overexpression, amplification, or mutation. These activating mechanisms offer potential therapeutic targets. Notably, the overexpression of MET and HGF in NSCLC is associated with a poor prognosis [38]. 

Crizotinib, known for its action as an ALK, ROS1, and MET inhibitor, was evaluated for its efficacy and safety in advanced NSCLC patients with MET amplification. It was found to be effective in MET-amplified NSCLC patients, presenting with a manageable adverse events profile. Interestingly, the ORR correlated with the level of MET amplification [39,40].

The intracranial effect of cabozantinib was evaluated in NSCLC patients with brain metastases and MET exon 14 skipping alterations, particularly in those who had developed brain metastases during crizotinib treatment. Cabozantinib demonstrated that it was effective in treating brain metastases in these cases [41]. 

On 3 February 2021, tepotinib was approved by the FDA for metastatic NSCLC with MET exon 14 skipping alterations. From the VISION trial, 152 advanced or metastatic NSCLC patients who presented with MET exon 14 skipping alterations received tepotinib orally once a day. Tepotinib showed a partial response in around half the patients with advanced NSCLC harboring MET exon 14 skipping [42].

On 10 August 2022, Capmatinib was also approved by the FDA for metastatic NSCLC with a mutation that leads to MET exon 14 skipping, detected using an FDA-approved test. This approval was based on the GEOMETRY mono-1 trial, and capmatinib in MET exon 14–mutated or MET-amplified NSCLC. Capmatinib demonstrated antitumor activity in advanced NSCLC patients harboring the MET exon 14 skipping mutation, with higher efficacy observed in those with a high gene copy number [43].

### 2.4. ROS1

ROS1 is a RTK belonging to the insulin receptor family, and it is known to be associated with chromosomal translocations in lung cancer. Extensive studies using NSCLC cell lines and patient-derived tumor samples have defined ROS fusions as attractive driver of mutations in NSCLC [44]. ROS rearrangements are more frequently observed in young never-smokers, females, those with a non-Asian ethnicity, and adenocarcinomas [45]. Given the genetic and clinical similarities between ALK-positive NSCLC and ROS-positive NSCLC, ALK-targeted therapies are often recommended as ROS1-targeted treatments.

Crizotinib and entrectinib are FDA-approved ROS1-targeted drugs for NSCLC patients. On 11 March 2016, based on data from the PROFILE 1001 trial [46], crizotinib was approved by the FDA as the first-line treatment for advanced (metastatic) NSCLC with an ROS1 gene alteration. It was the first and only FDA approved drug for ROS-1 positive NSCLC patients. The most common resistant mutations after crizotinib treatment include G2032R, D2033N, and S1986F [47]. 

Entrectinib, the second FDA-approved ROS1-targeted agent, targets not only ROS1 but also ALK and TRK in NSCLC. It possesses the ability to penetrate the blood–brain barrier, making it beneficial for patients with CNS metastases. On 15 August 2019, the FDA approved entrectinib for ROS1-positive metastatic NSCLC patients. This approval was based on ALKA, STARTRK-1, and STARTRK-2 trials [48,49].

Ceritinib is a second-generation ALK-TKI which also targets ROS1. It was approved by the FDA for ALK-positive metastatic NSCLC, but not for ROS1-positive NSCLC. In a phase II clinical trial conducted in South Korea, ceritinib showed potent activity in ROS1-rearranged NSCLC patients who were previously treated with multiple chemotherapies [50].

Lorlatinib, a highly selective, third-generation ALK-TKI, also functions as a ROS1 inhibitor. The FDA has approved lorlatinib for patients with ALK-positive metastatic NSCLC, but its use for ROS1-positive NSCLC is yet to be approved. Lorlatinib exhibits the ability to penetrate the CNS, and it is effective against various ROS1 resistance mechanisms [51].

Repotrectinib, a next-generation TKI for ROS1, TRK, and ALK, has shown promise in overcoming resistance caused by acquired ROS1, TRK, and ALK solvent-front mutations. In preclinical studies, repotrectinib demonstrated activity against ROS1 resistant mutations (G2032R and D2033N), and it displayed CNS-penetrating effects [52]. In the TRIDENT-1 clinical trial, the safety-related and preliminary effects of repotrectinib were evaluated in advanced ROS1-positive NSCLC patients, revealing that it was well-tolerated, and it exhibited potent clinical activity [53].

Another new-generation TKI, taletrectinib, targets TRK and ROS1 fusions. Taletrectinib was found to be effective in overcoming the G2032R mutation, which confers a high resistance against crizotinib [54]. In the first human study concerning taletrectinib, the drug demonstrated good tolerability, and efficacy signals were observed in ROS1-positive NSCLC patients [55].

### 2.5. BRAF

In accordance with the previous retrospective studies of NSCLC, BRAF mutations were identified at a 1–5% rate, mainly in adenocarcinomas. The most common BRAF mutation was V600E (valine to glutamate substitution at codon 600), followed by G469A and D594G. V600E mutations were frequently observed in females and current/former smokers [56,57]. The precise prevalence, distribution, and prognostic significance of BRAF mutations in lung cancer remain uncertain.

Due to the rarity of BRAF mutations, only a limited number of studies have investigated BRAF inhibitors like dabrafenib and vemurafenib. On 22 June 2017, the FDA granted approvals for a dabrafenib and trametinib combination to treat metastatic NSCLC patients with the BRAF V600E mutation. This approval was based on a clinical trial concerning BRF113928, and it was a phase II study of the BRAF inhibitor, dabrafenib. It was studied as a single agent, and in combination with the MEK inhibitor, trametinib, in subjects with BRAF V600E mutation positive metastatic NSCLC. It was the first FDA approval for BRAF V600E mutation-positive metastatic NSCLC treatment.

The French National Cancer Institute (INCa) performed a phase II clinical trial to identify the efficacy and safety of vemurafenib in NSCLC patients with BRAF^V600^- and BRAF^nonV600^ mutations. Out of the total 118 enrolled patients, 101 exhibited a BRAF^V600^ mutation, and 17 had BRAF^nonV600^ mutations. In this study, it was emphasized that vemurafenib is effective for BRAF^V600^-mutated NSCLC, but it is not effective for BRAF^nonV600^-mutated NSCLC. In addition, the authors also recommended routine biomarker screening for NSCLC, including BRAF^V600^ mutations [58]. There are also several ongoing clinical trials that concern a vemurafenib and cobimetinib combination to treat lung cancer. Vemurafenib is approved as the standard of care for unresectable or metastatic melanoma, and cobimetinib is approved as the standard of care in combination with vemurafenib for unresectable or metastatic melanoma. The phase II NAUTIKA1 study (NCT04302025) will include a BRAF cohort, and the participants will receive vemurafenib and cobimetinib as both a neoadjuvant treatment before surgery and an adjuvant therapy after surgery. In the phase II/III B-FAST trial (NCT03178552), the participants with the BRAF V600 mutation will receive atezolizumab, cobimetinib, and vemurafenib as a triple-combination therapy. The other phase II/III clinical trial, DETERMINE, is an umbrella-basket trial that will evaluate the efficacy of targeted therapies in common cancers with rare actionable genomic alterations. In this study, vemurafenib and cobimetinib will be used to treat BRAF positive cancer patients.

### 2.6. RET

RET is a receptor tyrosine kinase which plays a role in cellular proliferation, migration, and differentiation [59]. RET fusions are observed in approximately 1–2% of NSCLC patients, particularly in cases of adenocarcinomas or adenosquamous carcinomas [60]. Given that multi-targeted TKIs suppress RET kinase activity, these agents could be considered promising targeted therapies for lung cancer patients harboring RET fusions.

On 21 September 2022, selpercatinib was approved by the FDA for locally advanced or metastatic RET fusion-positive NSCLC. Prior to that, it was granted accelerated approval in 2020, based on the LIBRETTO-001 trial.

In addition, very recently, on 9 August 2023, the FDA granted approval to pralsetinib for RET gene fusion-positive NSCLC. The accelerated approval for pralsetinib was previously granted in 2020, based on the ARROW trial.

The phase II clinical trial, which treats RET fusion-positive NSCLC patients with cabozantinib, is ongoing (NCT01639508). Cabozantinib is known to inhibit RET, ROS1, NTRK, MET, and AXL. Therefore, RET fusion-positive advanced NSCLC patients with ROS1 or NTRK fusions, or increased MET or AXL activity, were enrolled in this study.

## 3. Colorectal Cancer

Colorectal cancer is a significant malignancy that primarily affects the colon and rectum, and it ranks among the leading causes of cancer-related deaths worldwide. Although highly developed countries have shown higher incidence rates, middle- and low-income countries are witnessing an increase in cases due to westernization. Particularly concerning is the noticeable rise in the incidence of early-onset colorectal cancer [61]. Despite advances in early detection and treatment, colorectal cancer often presents with nonspecific symptoms in its early stages, making a timely diagnosis challenging [62]. The improved understanding of colorectal cancer has resulted in a broader range of treatment options. These options encompass endoscopic and surgical excision, radiotherapy, chemotherapy, immunotherapy, and targeted therapy [63]. Targeted therapies have become an integral component for the treatment of metastatic colorectal cancer, leading to notable enhancements in survival outcomes.

Targeted therapies for colorectal cancer have been summarized in Table 2.

### 3.1. VEGF

VEGF, a critical angiogenic growth factor, plays a pivotal role in promoting endothelial cell survival, proliferation, and migration. In colorectal cancer treatment, some targeted drugs aim to inhibit VEGF function.

The AVF2107g trial has demonstrated the potential benefit of adding bevacizumab, a monoclonal antibody that blocks VEGF, to fluorouracil-based combination chemotherapy regimens in colorectal cancer. Eight-hundred and thirteen patients with metastatic colorectal cancer were treated with irinotecan, bolus fluorouracil, and leucovorin (IFL), plus bevacizumab or an IFL, plus a placebo, as a first-line therapy. The group with IFL plus bevacizumab showed an improved outcome with regard to the median duration of survival (20.3 months vs. 15.6 months), the median duration of PFS (10.6 months vs. 6.2 months), and the median duration of response (10.4 months vs. 7.1 months), as compared with the group that was treated with the IFL plus placebo [64]. In the E3200 study, bevacizumab was also found to be effective as a second-line treatment. Eight-hundred and twenty-nine metastatic colorectal cancer patients who were previously treated with a fluoropyrimidine and irinotecan combination received the following: (i) oxaliplatin, fluorouracil, and leucovorin (FOLFOX4) with bevacizumab; (ii) only FOLFOX4; or (iii) only bevacizumab. The patients treated with FOLFOX4 plus bevacizumab showed a prolonged duration of survival [65]. Bevacizumab was approved by the FDA for metastatic colorectal cancer in combination with (i) intravenous fluorouracil-based chemotherapy as a first- or second-line treatment, and (ii) fluoropyrimidine-irinotecan- or fluoropyrimidine-oxaliplatin-based chemotherapy as a second-line treatment in patients who have progressed when placed on a first-line bevacizumab product-containing regimen.

Ziv-aflibercept, a fully humanized, soluble recombinant fusion protein, effectively targets angiogenesis by inhibiting VEGF-A, VEGF-B, and the placental growth factor. Notably, it exhibits a superior binding affinity to VEGF-A compared with bevacizumab [66]. On 3 August 2012, based on phase III VELOUR study, the FDA approved ziv-aflibercept, in combination with 5-fluorouracil, leucovorin, and irinotecan (FOLFIRI), for metastatic colorectal cancer patients who are resistant to, or whose cancer has progressed, following an oxaliplatin-containing regimen. Metastatic colorectal cancer patients who were treated previously with an oxaliplatin-based regimen received ziv-aflibercept or a placebo in combination with FOLFIRI. The addition of ziv-aflibercept to FOLFIRI statistically improved survival over the placebo with FOLFIRI treatment [67].

Ramucirumab is a human IgG-1 monoclonal antibody that acts as a VEGF receptor 2 (VEGFR2) inhibitor. In the RAISE study, ramucirumab was compared against the placebo in combination with the second-line FOLFIRI treatment for metastatic colorectal carcinoma patients who progressed during or after first-line therapy with bevacizumab, oxaliplatin, and a fluoropyrimidine. Compared with the placebo plus FOLFIRI group, the ramucirumab with FOLFIRI group significantly prolonged overall survival when used as a second-line therapy for metastatic colorectal cancer [68]. On 24 April 2015, the FDA approved ramucirumab as a second-line treatment in combination with FOLFIRI for metastatic colorectal cancer patients whose cancer had progressed when placed on a first-line bevacizumab-containing regimen.

### 3.2. EGFR

EGFR belongs to the ErbB family of receptor tyrosine kinases. Upon ligand binding with its extracellular domain, the tyrosine kinase domain becomes phosphorylated, initiating signaling pathways responsible for cell proliferation, angiogenesis, migration, survival, and adhesion. Given the critical role of these pathways in cancer cell growth, EGFR represents a significant therapeutic target for the treatment of colorectal cancer metastases.

Cetuximab was approved by the FDA for K-Ras wild-type, EGFR-positive, metastatic colorectal cancer (i) in combination with FOLFIRI as a first-line treatment, (ii) in combination with irinotecan in patients who are refractory to irinotecan-based chemotherapy, and (iii) as a single agent in patients who have failed oxaliplatin- and irinotecan-based chemotherapy or who are intolerant to irinotecan. The efficacy of cetuximab against metastatic colorectal cancer was demonstrated in the BOND study, suggesting that targeting the EGFR pathway is key to overcoming chemotherapy resistance [69]. Cetuximab proved to be effective as a first-line treatment for EGFR-positive metastatic colorectal cancers, in combination with FOLFIRI, in a CRYSTAL trial [70]. A combination therapy comprising cetuximab and an oxaliplatin-based chemotherapy as a first-line treatment was also performed, however, benefits were not shown for this combination [71].

Another EGFR antagonist, panitumumab, has also been approved by the FDA for wild-type RAS metastatic colorectal cancer in combination with FOLFOX as a first-line treatment [72] and as a monotherapy after chemotherapy-derived disease progression. It is not recommended for RAS-mutant metastatic colorectal cancer patients, or for those with an unknown RAS mutation status.

### 3.3. BRAF

BRAF mutations, particularly the V600E mutation, in metastatic colorectal cancer are associated with a poor prognosis and reduced response, especially when treated with EGFR inhibitors. As a result, targeting this molecular abnormality has become a special focus in the treatment of metastatic colorectal cancer [73]. BRAF mutations in metastatic colorectal cancer are more frequently observed in older individuals (>70 years), current or former smokers, white patients, females, and those with KRAS wild-type tumors [74].

For patients with BRAFV600E–mutated metastatic colorectal cancer, combination targeted therapies are often adopted to increase therapeutic efficacy. The BEACON CRC study was conducted to evaluate the efficacy and safety of the combination of the BRAF inhibitor (encorafenib), MEK inhibitor (binimetinib), and EGFR inhibitor (cetuximab) in BRAFV600E–mutant metastatic colorectal cancer patients. This combination demonstrated promising clinical effects with a manageable safety profile, leading to the FDA approval of encorafenib. On 8 April 2020, the FDA approved encorafenib in combination with cetuximab for metastatic colorectal cancer patients with a BRAF V600E mutation.

Cetuximab exhibits several immunomodulatory effects, including antibody-dependent cellular cytotoxicity (ADCC), the induction of immunogenic cell death, and the promotion of immune cell infiltration. Moreover, previous studies have suggested a synergistic effect when cetuximab is combined with PD-1 monoclonal antibodies in colorectal cancer. Building upon these findings, a phase I clinical trial investigating the safety and preliminary efficacy of the BRAF inhibitor (vemurafenib) and EGFR inhibitor (cetuximab) regimen, combined with the PD-1 monoclonal antibody (camrelizumab) for BRAFV600E–mutant/MSS type metastatic colorectal cancer, is ongoing (NCT05019534). Another clinical study, IMPROVEMENT, is also ongoing for BRAFV600E–mutated advanced colorectal cancer patients. This phase II study evaluates the efficacy and safety of FOLFIRI when combined with the BRAF inhibitor (vemurafenib) and EGFR inhibitor (cetuximab) (NCT03727763).

### 3.4. TGF-β

Various researchers are currently directing their efforts towards the development of novel targeted agents, moving beyond the well-known targets. One such rare target is the Transforming Growth Factor-beta (TGF-β), which has not been considered as a favorable target for cancer treatment due to its dual role in promoting or inhibiting tumor growth, depending on the tumor stage and cellular context [75,76]. However, emerging studies, including those focusing on colorectal cancer, are demonstrating that targeting the TGF-β signaling pathway could effectively reduce cancer metastasis, making it a crucial target for drug development. Current anti-TGF-β therapies involve monoclonal antibodies or peptides targeting the ligand-receptor level and TGF-β receptor kinase inhibitors [77]. Although TGF-β inhibition has not yet been utilized in colorectal cancer treatment, extensive research is underway to explore various candidate molecules.

Galunisertib (LY2157299) is an orally administered small molecule inhibitor that selectively targets the kinase activity of the TGF-β receptor I (TβRI). It effectively reduces the phosphorylation of SMAD2, thereby disrupting the activation of the canonical pathway. Galunisertib has demonstrated anti-tumor efficacy in animal models of colon, breast, lung, and liver cancer [78]. Currently, a phase I/II clinical study combining galunisertib with capecitabine, in advanced chemotherapy-resistant colorectal cancer patients harboring peritoneal metastases (NCT05700656), is awaiting recruitment.

Another promising candidate is PF-03446962, a fully human IgG-2 monoclonal antibody targeting TβRI. Phase I and II clinical trials have been conducted with PF-03446962 in various cancer types, including advanced solid tumors [79], hepatocellular carcinoma [80], urothelial cancer (phase II trial) [81], advanced malignant pleural mesothelioma (phase II trial) [82], and refractory metastatic colorectal cancer (REGAL-1 trial) [83]. The REGAL-1 study aimed to evaluate the safety and tolerability of PF-03446962 in combination with regorafenib in refractory metastatic colorectal cancer patients. Although no meaningful clinical benefit was observed in this study, further research is required to determine whether the combination of a TGF-β inhibitor and an anti-angiogenic drug could be beneficial for refractory metastatic colorectal cancer patients.

### 3.5. Other Targets

HER2 amplifications are found in 1–4% of metastatic colorectal cancer patients [84]. On 19 January 2023, the FDA granted accelerated approval to tucatinib with trastuzumab for RAS wild-type, HER2-positive, unresectable or metastatic colorectal cancer patients who have progressed following fluoropyrimidine-, oxaliplatin-, and irinotecan-based chemotherapy. This treatment is the first FDA-approved anti-HER2 therapy for metastatic colorectal cancer. The accelerated approval was based on a phase II MOUNTAINEER study, and this trial is still ongoing [85].

MET overexpression has been found in colorectal cancer and it is known to be associated with cancer progression and metastasis [86]. Up until now, a range of MET-specific TKIs have demonstrated significant efficacy in clinical trials for various cancers, including NSCLC and gastric cancer. Nonetheless, there is still insufficient clinical trial data to assess the effectiveness of selective TKIs for treating colorectal cancer. Cabozantinib, a multi-targeted inhibitor of RTKs, targets MET, VEGFR2, AXL, RET, and ROS1. Its effectiveness against patients with refractory metastatic colorectal cancer was demonstrated in a phase II trial (NCT03542877). Building upon this clinical data and preclinical evidence from mouse colorectal cancer models, a new phase II clinical trial has been initiated. This trial investigates the combination of cabozantinib and nivolumab in patients with refractory metastatic microsatellite-stable colorectal cancer (NCT04963283).

The Wnt signaling pathway is another proposed target for colorectal cancer, however, as of yet, no promising results have been reported. Targeted compounds such as WNT974 (LGK974) and FOXY-5 have been tested to inhibit this pathway, with the expectation of demonstrating anti-tumor efficacy against colorectal cancer.

Immune checkpoint-targeted therapies amplify immune surveillance and suppression against cancer by inhibiting the tumor’s efforts to evade T cell detection [87]. Checkpoint inhibitors are under investigation across diverse solid tumors, and they have exhibited encouraging responses. In the context of metastatic colorectal cancer, despite notable responses observed in certain phase I trials, subsequent investigations revealed that only a limited number of colorectal cancer patients showed a positive reaction to immune checkpoint therapy. This specific subgroup of patients exhibited a high tumor mutational burden (TMB), alongside high levels of microsatellite instability (MSI-H), or a deficiency in mismatch repair (dMMR) in their tumor [88,89]. Pembrolizumab (PD-1 inhibitor) has gained approval for the treatment of unresectable or metastatic MSI-H or dMMR solid tumors. It received accelerated approval in 2017, which was later converted to full approval by the FDA on 29 March 2023. Similarly, nivolumab (PD-1 inhibitor) was granted accelerated approval by the FDA in 2017 for MSI-H or dMMR metastatic colorectal cancer. Furthermore, in 2018, the FDA provided accelerated approval for the use of ipilimumab (CTLA-4 inhibitor) in combination with nivolumab for treating patients with MSI-H or dMMR metastatic colorectal cancer. This approval led to a new indication being added to the nivolumab labeling.

## 4. Prostate Cancer

Prostate cancer poses a significant health burden, ranking as the most commonly diagnosed cancer and the second leading cause of cancer-related deaths in men in the United States [90]. It is also the most prevalent cancer among men in certain regions of Europe and Africa, and its incidence has been increasing in recent times [91]. Despite extensive research, the etiology and pathogenesis of prostate cancer remain poorly understood and complex. Several factors, including age, obesity, family history, and specific gene mutations (e.g., BRCA1 and BRCA2), have been linked to prostate cancer, but the precise mechanisms underlying its development are still unclear. The treatments for prostate cancer include surgery, radiation, hormonal therapy, and targeted therapy [92]. Emerging targeted therapies focus on specific molecular alterations in prostate cancer cells, offering potential benefits with fewer side effects, as compared with traditional treatments.

Targeted therapies for prostate cancer have been summarized in Table 3.

### 4.1. Androgen Receptor

The AR signaling pathway plays a crucial role in prostate cancer growth and survival. Antiandrogen compounds can directly inhibit the AR pathway by disrupting the binding of androgens to the AR. In prostate cancer cells, dihydrotestosterone (DHT) activates proteins that translocate to the nucleus, where they facilitate the transcription of genes that are responsible for regulating cell growth and survival. Antiandrogens like enzalutamide and apalutamide bind to the AR in the cytoplasm, preventing the interaction between androgens and the AR. This blocks the translocation of the AR into the nucleus, and subsequently, DNA binding [93]. Enzalutamide may additionally interfere with the coactivator function at the DNA binding site, further inhibiting transcription [94].

As standalone therapies, antiandrogens block the binding of DHT to the AR, but they do not significantly reduce serum testosterone levels. They are commonly used in combination with luteinizing hormone-releasing hormone (LHRH) agonists to mitigate the clinical impact of the initial testosterone surge. In men with metastatic prostate cancer, antiandrogens are also administered with a LHRH agonist or antagonist to achieve “complete androgen blockade” (CAB) [95]. Some clinicians prescribe antiandrogens as a monotherapy for patients with nonmetastatic disease who wish to avoid the metabolic effects of androgen deprivation therapy (ADT) [96].

In terms of ADT, there are first- and second-generation options, including antiandrogens and drugs that target the LHRH receptor. Third-generation drugs encompass a broader range of mechanisms, and they are collectively referred to as androgen pathway inhibitors.

Enzalutamide is an oral, nonsteroidal anti-androgen prescribed for metastatic castration-resistant prostate cancer (mCRPC). It competitively binds to the AR at the androgen-binding site, inhibiting nuclear translocation and interactions with DNA. Enzalutamide effectively blocks the cellular actions of testosterone, and it is administered in combination with ongoing ADT. A study comparing enzalutamide and placebo treatments, in mCRPC patients who had previously received chemotherapy, showed an improvement in median overall survival (18.4 months vs. 13.6 months) [97]. In chemotherapy-naïve patients, the estimated median overall survival for enzalutamide was not reached, with 82% of patients remaining alive at 18 months, compared with a median overall survival of 31.0 months in the placebo group [98]. In terms of safety, in addition to the anticipated adverse events associated with AR inhibitors, rare occurrences of seizures and posterior reversible encephalopathy syndrome have been reported, which is likely due to the drug crossing the blood–brain barrier. The most common adverse reactions include fatigue, hypertension, rash, and diarrhea. Enzalutamide is approved by the FDA for both castration-resistant prostate cancer and metastatic castration-sensitive prostate cancer.

Apalutamide, another oral nonsteroidal anti-androgen, targets the ligand-binding domain of the AR, and it is used to treat non-metastatic castration-resistant prostate cancer (nmCPRC), further validating the benefits of androgen pathway inhibition throughout the disease continuum [99]. In a study involving patients with nmCPRC, apalutamide in combination with ADT or a bilateral orchiectomy, demonstrated a metastasis-free survival of 40.5 months, compared with 16.2 months in the placebo group. The most common adverse events observed were fatigue, hypertension, rash, and diarrhea [100]. Apalutamide was initially approved by the FDA in 2018 for patients with nmCPRC. Subsequently, in 2019, it received another FDA approval for the treatment of metastatic castration-sensitive prostate cancer (mCSPC) in patients.

Darolutamide is an orally-administered, nonsteroidal anti-androgen with a similar mechanism of action to enzalutamide and apalutamide. On 30 July 2019, based on the ARAMIS trial, the FDA approved darolutamide for nmCPRC treatment. In the ARAMIS study, 1509 nmCPRC patients received either darolutamide or a placebo, and the darolutamide group exhibited significantly prolonged metastasis-free survival [101]. In addition, on 5 August 2022, darolutamide in combination with docetaxel was approved by the FDA for metastatic hormone-sensitive prostate cancer (mHSPC). This approval was based on the ARASENS trial comprising 1306 mHSPC patients. In this study, treatment with darolutamide plus docetaxel demonstrated a statistically significant delay in time-to-pain progression, as compared with darolutamide plus a placebo [102]. Ongoing research is exploring the use of darolutamide with combinations of various agents to improve treatment outcomes for prostate cancer patients.

### 4.2. CYP17

Targeting the enzymes involved in testosterone biosynthesis presents an additional therapeutic avenue for the management of prostate cancer. Testosterone, a steroid hormone derived from cholesterol through a series of biochemical reactions [103], undergoes its final biosynthesis steps which are facilitated via the enzyme, CYP17. It plays a crucial role in converting precursors, like pregnenolone, into the weaker androgen, dehydroepiandrosterone (DHEA), and progesterone into androstenedione. Normally, this enzyme is found in the testes and adrenal glands. However, certain prostate cancer cells can produce it and generate testosterone outside the normal regulatory mechanisms. By effectively inhibiting this enzyme, the biosynthesis of testosterone can be prevented across various sites, offering a potential strategy to counter the androgen-driven growth of prostate cancer cells.

Abiraterone acetate is an oral inhibitor of androgen biosynthesis that specifically targets the CYP17 enzyme, effectively blocking its enzymatic activity and inhibiting testosterone production [104]. It is typically used in combination with prednisone and ADT. Abiraterone exerts its effect by significantly reducing androgen production in various sources, including the testes, adrenal glands, and prostate cancer cells. Studies have demonstrated that when abiraterone is combined with ADT, testosterone suppression reaches lower levels compared with treatment that only uses an LHRH agonist [105]. In addition to its use in castration-resistant prostate cancer, abiraterone has also shown activity in other AR-positive malignancies, such as salivary gland carcinomas, when combined with LHRH analogs [106].

Recently, the LATITUDE trial evaluated the use of abiraterone in men with high-risk, metastatic, castration-sensitive prostate cancer, leading to its recent FDA approval as a treatment. The trial results revealed that abiraterone increased the overall survival rate and it showed improvements for several secondary clinical endpoints [107]. Similarly, the STAMPEDE trial investigated abiraterone in a similar patient group, including those with a node-positive disease and high-risk locally advanced prostate cancer. Although radiation therapy was essential for high-risk patients, it was optional for those with a node-positive disease. The results confirmed that abiraterone significantly reduced the number of deaths [108].

The combination of abiraterone with ADT achieves a dual and complementary pathway of testosterone suppression, leading to improved outcomes in patients with advanced prostate cancer [108,109,110]. It is important to note that even when testosterone levels are below 20 ng/dL, the continued stimulation of prostate cancer cell growth due to testosterone necessitates the suppression of testosterone to near-zero levels, which may provide additional benefits to patients with advanced prostate cancer.

As expected, there are adverse events caused by testosterone inhibition. Abiraterone may be associated with mineralocorticoid toxicity, including hypertension, hypokalemia, and fluid retention, as well as liver function abnormalities, some of which can be severe, such as fulminant hepatitis and acute liver failure. Therefore, it is important to closely monitor serum transaminases and bilirubin levels before initiating treatment, every 2 weeks for the first 3 months, and monthly thereafter. Blood pressure and potassium levels should also be monitored monthly. Additionally, the concurrent administration of prednisone may lead to adverse events such as confusion, restlessness, excitement, nausea, headache, and vomiting [111]. The careful monitoring and management of these adverse events are essential for optimal patient care.

The FDA initially approved abiraterone acetate in combination with prednisone in 2011 for mCRPC patients who had previously received chemotherapy. Subsequently, the indication was expanded in 2012 for mCRPC patients who had not received prior chemotherapy, making it an option for both pre- and post-chemotherapy patients with mCRPC. Additionally, in 2018, abiraterone acetate, in combination with prednisone, was approved for the treatment of metastatic high-risk castration-sensitive prostate cancer, further broadening its therapeutic use in prostate cancer management.

### 4.3. PARP

The presence of both endogenous and exogenous base DNA damage increases the risk of cancer by causing genomic instability. DNA damage response (DDR) involves several mechanisms that depend on the type of damage. DNA repair pathways, including base excision repair, nucleotide excision repair, homologous recombination repair (HRR), non-homologous end joining (NHEJ), translesion synthesis, and mismatch repair (MMR), play crucial roles in maintaining the integrity of the genome [112,113]. Among various types of DNA damage, single-strand breaks are the most common, and they are repaired via base-excision mechanisms involving PARP enzymes. Notably, PARPs are also activated in the repair of double-strand breaks during HRR. PARP enzymes serve as DNA damage sensors, and they catalyze the transfer of ADP-ribose to target proteins by cleaving NAD+ in the poly ADP-ribosylation reaction, which activates the DNA repair machinery. Additionally, PARP influences chromatin structure, DNA replication, and transcription [114]. In summary, DNA repair pathways, including the involvement of PARP enzymes, are critical for maintaining genomic stability and preventing cancer development.

AR that is located within the cell nucleus are crucial for governing gene expression in prostate biology [115], encompassing genes like the prostate specific antigen (PSA) and TMPRSS2. In the context of prostate cancer treatment, blocking androgen signaling is of utmost importance. ARs collaborate with co-repressors and co-stimulators, such as GATA2, HOXB13, and NKX3, to regulate transcription, thus contributing to the development of prostate cancer. FOXA1 also interacts with ARs, thus influencing their activity, and the increased expression of FOXA1 has been associated with a less favorable prognosis in prostate cancer patients. PARP2 enhances AR activity by interacting with FOXA1, whereas PARP1 deficiency in tumors leads to decreased levels of nuclear androgen receptors. Acting as transcription regulators of AR, the inhibition of the PARPs results in the reduced expression of AR-related genes. The genetic or pharmacological inhibition of PARP2-FOXA1 interactions hampers AR activity and impedes tumor growth [116,117]. Additionally, ARs participate in promoting the DNA damage response and regulating DNA repair genes, such as HR, NHEJ, and MMR. Antiandrogen therapy reduces HR gene expression and ATM signaling [118,119,120].

The relationship between cancer and the tumor microenvironment has become a significant issue in prostate pathobiology. PARP influences reciprocal interactions within the tumor microenvironment, impacting prostate tumorigenesis and aggressiveness [121,122,123,124,125,126]. Studies have shown higher expressions of PARP1 and PARP2 in prostate cancer compared with benign tissues, and PARP2 expression correlates with biochemical recurrence. The PARP’s role in the tumor microenvironment affects cancer-initiating cells, immune response, angiogenesis, autophagy, and the hypoxic response, thus influencing the development and growth of cancer, particularly in CRPC with alterations in HRR genes under hypoxic conditions [127,128].

The ETS family of transcription factors plays a crucial role in regulating cell migration and proliferation [129]. Gene rearrangements that increase ETS system activity contribute to cancer growth and development, including the fusion between TMPRSS2 and ERG, which is found in approximately 50% of prostate cancer cases. The overexpression of ETS genes leads to DNA double-strand breaks, and PARP and DNA-dependent protein kinase modulate the transcriptomic activity of the ETS system [128,129,130]. 

Olaparib has been shown to reduce the potential of invasion in ERG-positive prostate cancer cell lines through the inhibition of invasion-associated genes, for example, EZH2, which may provide new molecular therapeutic targets [128]. Despite promising results from preclinical studies, ETS stratification in clinical trials with abiraterone and veliparib did not confirm the clinical utility of ETS as a predictive factor [131]. Molecular stratification, based on ETS status and PTEN expression in prostate cancer, may better predict clinical outcomes in the localized and metastatic stages of disease [132,133]. TMPRSS2-ERG-positive and PTEN-negative tumors are more sensitive to PARP inhibitor and radiation therapy [134]. On 31 May 2023, the FDA granted approval for olaparib in combination with abiraterone and prednisone (or prednisolone) for the treatment of mCRPC in patients with deleterious or suspected deleterious BRCA mutations. Prior to this approval, olaparib was already approved for the treatment of mCRPC patients with deleterious, suspected deleterious germline, or somatic HRR gene mutations who had experienced disease progression after receiving enzalutamide or abiraterone.

Rucaparib was highly effective in men with mCRPC and BRCA alterations, as it led to significant improvements in radiographic and PSA responses, including complete responses in soft-tissue disease [135,136]. The treatment’s safety profile was manageable, resulting in accelerated approval by the US FDA for men with mCRPC and deleterious BRCA mutations who had prior AR-directed therapy and taxane-based chemotherapy. The baseline characteristics of the efficacy population were as expected for mCRPC patients undergoing a third-line or later treatment, with a notable proportion receiving next-generation AR-directed therapies [137,138].

The TALAPRO-1 trial demonstrated the effectiveness of talazoparib in treating mCRPC, particularly in men with BRCA1/2 alterations. The safety profile of talazoparib in heavily pretreated men with mCRPC and DDR-HRR alterations was detailed to guide dosing in future clinical trials and to provide clinicians with insights into managing treatment-related adverse events. The phase III EMBRACA and phase II ABRAZO trials established a similar safety profile for talazoparib in female and male patients with advanced breast cancer and gBRCA1/2 alterations, with common hematologic and non-hematologic adverse events reported. The TALAPRO-1 trial included older men who had received prior chemotherapy regimens for mCRPC [136]. Recently, on 20 June 2023, the FDA approved talazoparib in combination with enzalutamide for the treatment of mCRPC in patients with HRR gene mutations.

A new, highly selective PARP inhibitor, niraparib, has been submitted to the FDA as a new drug application (NDA); it is seeking approval as a first-line targeted treatment for mCRPC patients with BRCA mutations. It is intended to be used in combination with abiraterone acetate in the form of a dual-action tablet (DAT), along with prednisone. If approved, this will be the first DAT formulation available in the US for mCRPC patients with BRCA mutations, which represent a type of HRR gene alteration. The unique DAT formulation targets both the AR axis and HRR gene alterations in mCRPC patients. The NDA is based on the MAGNITUDE study, which evaluated the safety and efficacy of niraparib with abiraterone acetate, plus prednisone, as a first-line treatment for mCRPC patients, regardless of their HRR alteration status [139].

### 4.4. Other Targets

The prostate-specific membrane antigen (PSMA) is a transmembrane glycoprotein on the cell membrane. It is known to be highly expressed in prostate cancer, and its expression level is closely associated with tumor invasiveness. As such, PSMA has been under investigation as a potential molecular target for prostate cancer [140]. ^177^Lu-PSMA-617 is the most extensively investigated targeted drug for radioligand therapy in prostate cancer. This compound is created by conjugating the small molecule inhibitor PSMA-617 with the β-emitting radionuclide, ^177^Lu. ^225^Ac-PSMA-617 (PSMA-617 with α-emitting radionuclide ^225^Ac) and ^177^Lu-J591 (^177^Lu with monoclonal antibody J591) are also currently under investigation as potential radioligand therapies for prostate cancer.

Angiogenesis, another target for prostate cancer, forma new vascular vessels using existing blood vessels. It plays a crucial role in wound healing and embryonic development, as well as in facilitating the creation of collateral pathways to enhance organ perfusion in cases of ischemia [141]. Given the significant importance of angiogenesis in tumor progression, it is now recognized as one of the more promising targets for targeted cancer therapies. By binding to its receptors, VEGF-A triggers the activation of the RAS–RAF–MAPK (mitogen-activated protein kinase) signaling pathway, thereby promoting the proliferation of endothelial cells [142]. Due to the observed overexpression of VEGF-A in prostate cancer, coupled with its correlation with an unfavorable prognosis and metastasis, the majority of clinical investigations into anti-angiogenic approaches for prostate cancer have revolved around the inhibition of VEGF-A [143]. Unfortunately, the addition of an anti-angiogenic therapy (bevacizumab, aflibercept, sunitinib, and lenalidomide) to chemotherapy or hormonal treatment in refractory, castration-resistant prostate cancer, does not yield any clinical benefits, but it does increase toxicity [144,145,146,147,148,149].

Although immunotherapy has broadened treatment horizons for various cancers, leading to improved overall survival, its definitive significance within advanced prostate cancer cases remains unestablished, apart from limited retrospective studies involving dMMR, MSI-H, and cyclin-dependent kinase 12 (CDK12) patients. Prostate cancers with dMMR genes, MSI-H, and CDK12 biallelic alterations are reported to be associated with higher TMB and neoantigen loads, which may enhance antitumor immunity [150]. In mCRPC, immune checkpoint inhibitors (ICIs) have been extensively tested, but a monotherapy combined with anti-PD-1 or anti-CTLA-4 have not consistently shown overall survival benefits. Combinations of ICIs with standard treatments, like chemotherapy and targeted therapies, particularly in patients with specific genetic variations, have shown promise. Trials are now focusing on specific patient groups based on biomarkers like homologous recombination deficiency (HRD)-positive, CDK12-inactivated, and MSI-H tumors. These markers could guide patient selection in the evolving landscape of advanced prostate cancer.

## 5. Conclusions

In conclusion, targeted therapies have emerged as a promising approach for the treatment of various cancers, including lung cancer, colorectal cancer, and prostate cancer. The identification of specific molecular targets in cancer cells has led to the development of a diverse range of targeted drugs that selectively inhibit key pathways involved in tumor growth and progression. Overall, targeted therapies have revolutionized cancer treatments, offering the potential for personalized and more effective approaches. However, challenges such as resistance mechanisms, biomarker identification, and optimal combination strategies still require attention to further enhance the efficacy and clinical impact of targeted drugs. Furthermore, it should be noted that certain individuals may display am exceptional sensitivity to targeted therapies, resulting in specific and severe adverse effects. Continued research and collaboration between academic institutions, industries, and regulatory authorities will be crucial in advancing the field of targeted therapies, and ultimately, improving cancer patients’ outcomes.

## Figures and Tables

**Table 1:** Targeted therapies for lung cancer.

Target	Alteration	Frequency	Targeted Agent	Status
EGFR-sensitizing	Mutation	17%	(First generation EGFR-TKIs)	
			Gefitinib	Approved by FDA
			Erlotinib	Approved by FDA
			(Second generation EGFR-TKIs)	
			Afatinib	Approved by FDA
			Dacomitinib	Approved by FDA
			(Third generation EGFR-TKIs)	
			Osimertinib	Approved by FDA
ALK	Rearrangement	7%	(First generation ALK-TKIs)	
			Crizotinib	Approved by FDA
			(Second generation ALK-TKIs)	
			Alectinib	Approved by FDA
			Ceritinib	Approved by FDA
			Brigatinib	Approved by FDA
			(Third generation ALK-TKIs)	
			Lorlatinib	Approved by FDA
MET	Alteration	3%	Tepotinib	Approved by FDA
			Capmatinib	Approved by FDA
			Crizotinib	Phase II
			Cabozantinib	Phase II
ROS1	Rearrangement	2%	Crizotinib	Approved by FDA
			Entrectinib	Approved by FDA
			Ceritinib	Phase II
			Lorlatinib	Phase II
			Repotrectinib	Phase I/II
			Taletrectinib	Phase II
BRAF	Mutation	1–5%	Dabrafenib (with Trametinib)	Approved by FDA
			Vemurafenib	Phase II/III
RET	Rearrangement	1–2%	Selpercatinib	Approved by FDA
			Pralsetinib	Approved by FDA
			Cabozantinib	Phase II

**Table 2:** Targeted therapies for colorectal cancer.

Target	Targeted Agent	Status	Indications and Usage/Study Title
VEGF	Bevacizumab	Approved by FDA	Used to treat metastatic colorectal cancer, in combination with intravenous fluorouracil-based chemotherapy as a first- or second-line treatment. Used to treat metastatic colorectal cancer, in combination with fluoropyrimidine-irinotecan- or fluoropyrimidine-oxaliplatin-based chemotherapy as a second-line treatment in patients whose cancer progressed when placed on a first-line bevacizumab product-containing regimen.
	Ziv-aflibercept	Approved by FDA	Used to treat metastatic colorectal cancer that is resistant to, or has progressed, when treated with an oxaliplatin-containing regimen in combination with FOLFIRI.
	Ramucirumab	Approved by FDA	Used to treat metastatic colorectal cancer when the disease has progressed during or after prior therapy with bevacizumab, oxaliplatin, and fluoropyrimidine, in combination with FOLFIRI.
EGFR	Cetuximab	Approved by FDA	Used to treat K-Ras wild-type, EGFR-expressing, metastatic colorectal cancer, as determined using an FDA-approved test. Used in combination with FOLFIRI as a first-line treatment. Used in combination with irinotecan in patients who are refractory to irinotecan-based chemotherapy. Used as a single agent in patients who have failed oxaliplatin- and irinotecan-based chemotherapy, or who are intolerant to irinotecan.
	Panitumumab	Approved by FDA	Used to treat wild-type RAS (defined as wild-type in both KRAS and NRAS, as determined using an FDA-approved test for this use) metastatic colorectal cancer. Used in combination with FOLFOX as a first-line treatment. Used as a monotherapy following disease progression after prior treatment with fluoropyrimidine, oxaliplatin, and an irinotecan-containing chemotherapy. Limitations of use include the fact that it is not recommended for the treatment of patients with RAS-mutant metastatic colorectal cancer, or for whom RAS mutation status is unknown.
BRAF	Encorafenib	Approved by FDA	Used to treat metastatic colorectal cancer with a BRAF V600E mutation, in combination with cetuximab, after prior therapy. Limitations of use include the fact that it is not recommended for the treatment of patients with wild-type BRAF colorectal cancer.
	Vemurafenib	Phase I	A Phase I Study on the Tolerance and Safety of Vemurafenib Film-coated Tablets and a Cetuximab Solution for the Infusion and Camrelizumab Protocol (VCC) concerning the After Line Therapy for BRAF V600E Mutation/MSS Metastatic Colorectal Cancer (NCT05019534).
		Phase II	Cetuximab and Vemurafenib Plus FOLFIRI for BRAF V600E Mutated Advanced Colorectal Cancer (IMPROVEMENT): A Single-arm Study (NCT03727763).
TβRI	Galunisertib (LY2157299)	Phase I/II	Phase I/II Study concerning Galunisertib Combined With Capecitabine in Patients With Advanced Chemotherapy-Resistant Colorectal Cancer With Peritoneal Metastases (NCT05700656).
	PF-03446962	Phase I	Phase Ib Study concerning the Combination of Regorafenib With PF-03446962 in Patients With Refractory Metastatic Colorectal Cancer (REGAL-1 Trial) (NCT02116894).
HER2	Tucatinib with Trastuzumab	Approved by FDA	Used to treat RAS wild-type HER2-positive unresectable or metastatic colorectal cancer that has progressed following treatment with fluoropyrimidine-, oxaliplatin-, and irinotecan-based chemotherapy.
MET	Cabozantinib	Phase II	A Phase II Study of Cabozantinib and Nivolumab in Refractory Metastatic Microsatellite Stable (MSS) Colorectal Cancer (NCT04963283).

**Table 3:** Targeted therapies for prostate cancer.

Target	Targeted Agent	Status	Indications and Usage/Study Title
Androgen Receptor	Enzalutamide	Approved by FDA	Used to treat castration-resistant prostate cancer. Used to treat metastatic castration-sensitive prostate cancer.
	Apalutamide	Approved by FDA	Used to treat metastatic castration-sensitive prostate cancer (mCSPC). Used to treat non-metastatic castration-resistant prostate cancer (nmCRPC).
	Darolutamide	Approved by FDA	Used to treat non-metastatic castration-resistant prostate cancer (nmCRPC) Used to treat metastatic hormone-sensitive prostate cancer (mHSPC) in combination with docetaxel.
CYP17	Abiraterone acetate	Approved by FDA	Used in combination with prednisone to treat the following: metastatic castration-resistant prostate cancer (mCRPC); metastatic high-risk castration-sensitive prostate cancer (CSPC).
PARP	Olaparib	Approved by FDA	Used to treat deleterious or suspected deleterious germline or somatic HRR gene-mutated metastatic castration-resistant prostate cancer (mCRPC), which has progressed following prior treatment with enzalutamide or abiraterone. Used to treat deleterious or suspected deleterious BRCA-mutated (BRCAm) metastatic castration-resistant prostate cancer (mCRPC), in combination with abiraterone and prednisone or prednisolone.
	Rucaparib	Approved by FDA	Used to treat deleterious BRCA mutation (germline and/or somatic)-associated metastatic castration-resistant prostate cancer (mCRPC) which has been treated with an androgen receptor-directed therapy and a taxane-based chemotherapy.
	Talazoparib	Approved by FDA	Used to treat HRR gene-mutated metastatic castration-resistant prostate cancer (mCRPC), in combination with enzalutamide.
	Niraparib	NDA	Used to treat BRCA-positive mCRPC, in combination with abiraterone acetate, in the form of a dual-action tablet (DAT), plus prednisone.
PSMA	^177^Lu-PSMA-617	Phase III	VISION: An International, Prospective, Open Label, Multicenter, Randomized Phase 3 Study of ^177^Lu-PSMA-617 in the Treatment of Patients With Progressive PSMA-positive Metastatic Castration-resistant Prostate Cancer (mCRPC) (NCT03511664).
		Phase III	PSMAfore: A Phase III, Open-label, Multi-Center, Randomized Study Comparing ^177^Lu-PSMA-617 vs. a Change in Androgen Receptor-directed Therapy in the Treatment of Taxane Naïve Men With Progressive Metastatic Castrate Resistant Prostate Cancer (NCT04689828).
		Phase III	An Open-label, Randomized, Phase III Study Comparing ^177^Lu-PSMA-617 in Combination With Standard of Care, Versus Standard of Care Alone, in Adult Male Patients With Metastatic Hormone Sensitive Prostate Cancer (mHSPC) (NCT04720157).
		Phase II	UpFrontPSMA: A Randomised Phase 2 Study of Sequential ^177^Lu-PSMA617 and Docetaxel Versus Docetaxel in Metastatic Hormone-Naive Prostate Cancer (NCT04343885).
		Phase II	ENZA-p: A Randomised Phase II Trial Using PSMA as a Therapeutic Agent and Prognostic Indicator in Men With Metastatic Castration-resistant Prostate Cancer Treated With Enzalutamide (ANZUP 1901) (NCT04419402).
		Phase II	Lutetium-177-PSMA-617 Radioligand Therapy in Oligo-metastatic Hormone Sensitive Prostate Cancer (NCT04443062).
		Phase I/II	Study of the Dosimetry, Safety, and Potential Benefits of ^177^Lu-PSMA-617 Radionuclide Therapy Prior to Radical Prostatectomy in Men With High-risk Localised Prostate Cancer (NCT04430192).
		Phase I	Immunogenic Priming With PSMA-Targeted Radioligand Therapy in Advanced Prostate Cancer: A Phase 1b Study of ^177^Lu-PSMA-617 in Combination With Pembrolizumab (NCT03805594).
		Phase I	^177^Lu-PSMA-617 Therapy and Olaparib in Patients With Metastatic Castration Resistant Prostate Cancer (NCT03874884).
	^225^Ac-PSMA-617	Phase I	AcTION: A Phase I Study of [^225^Ac]Ac-PSMA-617 in Men With PSMA-positive Prostate Cancer With or Without Prior [^177^Lu]Lu-PSMA-617 Radioligand Therapy (NCT04597411).
	^177^Lu-J591	Phase II	A Randomized Phase II Trial of ^177^Lu Radiolabeled Monoclonal Antibody HuJ591 (^177^Lu-J591) and Ketoconazole in Patients With High-Risk Castrate Biochemically Relapsed Prostate Cancer After Local Therapy (NCT00859781).

## Data Availability

Data sharing not applicable.

## Abbreviations

NSCLC	non-small cell lung cancer
EGFR	epidermal growth factor receptor
TKI	tyrosine kinase inhibitor
PFS	progression-free survival
FGFR	fibroblast growth factor receptor
IGF1R	insulin-like growth factor 1 receptor
AXL	anexelekto
ALK	anaplastic lymphoma kina
EML4	echinoderm microtubule-associated protein-like 4
MET	mesenchymal-epithelial transition
ORR	objective response rate
KIT	mast/stem cell growth factor receptor
RTK	receptor tyrosine kinase
HGF	hepatocyte growth factor
FDA	Food and Drug Administration
VEGF	vascular endothelial growth factor
IFL	irinotecan, bolus fluorouracil, and leucovorin
FOLFOX4	oxaliplatin, fluorouracil, and leucovorin
FOLFIRI	5-fluorouracil, leucovorin, and irinotecan
VEGFR	VEGF receptor
ADCC	antibody-dependent cellular cytotoxicity
TGF-β	transforming growth factor-beta
TβRI	TGFβ receptor I
HER2	human epidermal growth factor receptor 2
TMB	tumor mutational burden
MSI-H	microsatellite instability-high
dMMR	deficient mismatch repair
AR	androgen receptor
DHT	dihydrotestosterone
LHRH	luteinizing hormone-releasing hormone
CAB	complete androgen blockade
ADT	androgen deprivation therapy
DHEA	dehydroepiandrosterone
mCRPC	metastatic castration-resistant prostate cancer
nmCRPC	non-metastatic castration-resistant prostate cancer
mHSPC	metastatic hormone-sensitive prostate cancer
mCSPC	metastatic castration-sensitive prostate cancer
HRR	homologous recombination repair
DDR	DNA damage response
NHEJ	non-homologous end joining
MMR	mismatch repair
PSA	prostate specific antigen
NDA	new drug application
PSMA	prostate-specific membrane antigen
MAPK	mitogen-activated protein kinase
CDK12	cyclin-dependent kinase 12
ICI	immune checkpoint inhibitor
HRD	homologous recombination deficiency
